# The physical activity health paradox: what is it, why might it happen, and where to go from here?

**DOI:** 10.1186/s12966-026-01922-z

**Published:** 2026-05-08

**Authors:** Tyler D. Quinn, Stephanie A. Prince, Nicolaas P. Pronk, Bethany Barone Gibbs

**Affiliations:** 1 Department of Epidemiology and Biostatistics, West Virginia University School of Public Health, Morgantown, WV USA; 2 Centre for Surveillance and Applied Research, Public Health Agency of Canada, Ottawa, ON Canada; 3 School of Epidemiology and Public Health, Faculty of Medicine, University of Ottawa, Ottawa, ON Canada; 4 HealthPartners Institute US, Bloomington, MN USA; 5 School of Public Health, University of Minnesota, Minneapolis, MN USA

**Keywords:** Occupation, Workplace, Exercise, Cardiovascular, Epidemiology, Sedentary behavior

## Abstract

**Background:**

The “physical activity health paradox” posits that physical activity done during work (occupational physical activity [OPA]) may not yield the health benefits consistently observed for leisure-time physical activity (LTPA) and, in some cases, may be harmful. Given the broad implications for such a paradox, which contradicts current public health guidelines for physical activity, we conducted a narrative, non-systematic review to discuss the current epidemiological and mechanistic evidence on the topic to inform opportunities for research and practice moving forward.

**Epidemiological evidence:**

Epidemiological evidence shows that LTPA is reliably protective against mortality and cardiovascular disease, whereas OPA has mixed or adverse associations. Several recent meta-analyses found higher all-cause mortality risk among men with high vs low OPA and found LTPA to potentially mitigate this OPA risk. Studies with device-measured OPA further highlight potential heterogeneity by OPA task and context. These conclusions remain limited by low quality evidence due to heterogeneous OPA exposure measurements, referent group selection, challenges in study design, and varied confounder adjustments.

**Mechanistic evidence:**

Mechanistically, four interrelated pathways that may explain the observed presence of a paradox have been proposed and preliminarily tested: (1) acute cardiovascular strain catalyzed by long-duration OPA with little recovery; (2) downstream vascular changes such as greater arterial stiffness, blunted baroreflex sensitivity, and maladaptive cardiac remodeling from chronic OPA exposure; (3) systemic inflammation associated with high OPA levels; and (4) modifiers such as low cardiorespiratory fitness and high psychosocial stress amplifying strain, inflammation, and risk. Current evidence is limited by reliance on cross-sectional or between-subject designs, crude OPA classification, and limited mechanistic interventions.

**Conclusions:**

Unlike the clear benefits from LTPA, research findings examining the health effects of OPA remain mixed. While uncertainty remains, the balance of evidence suggests that OPA is less beneficial to health than LTPA which should be considered in public health messaging. Advancing the field will require multidimensional OPA exposure assessment, rigorous study designs, and evaluation of mechanism-driven outcomes to clarify causal pathways and identify feasible intervention targets to promote health in workers with physically demanding jobs.

## Introduction

**Fig. 1:**
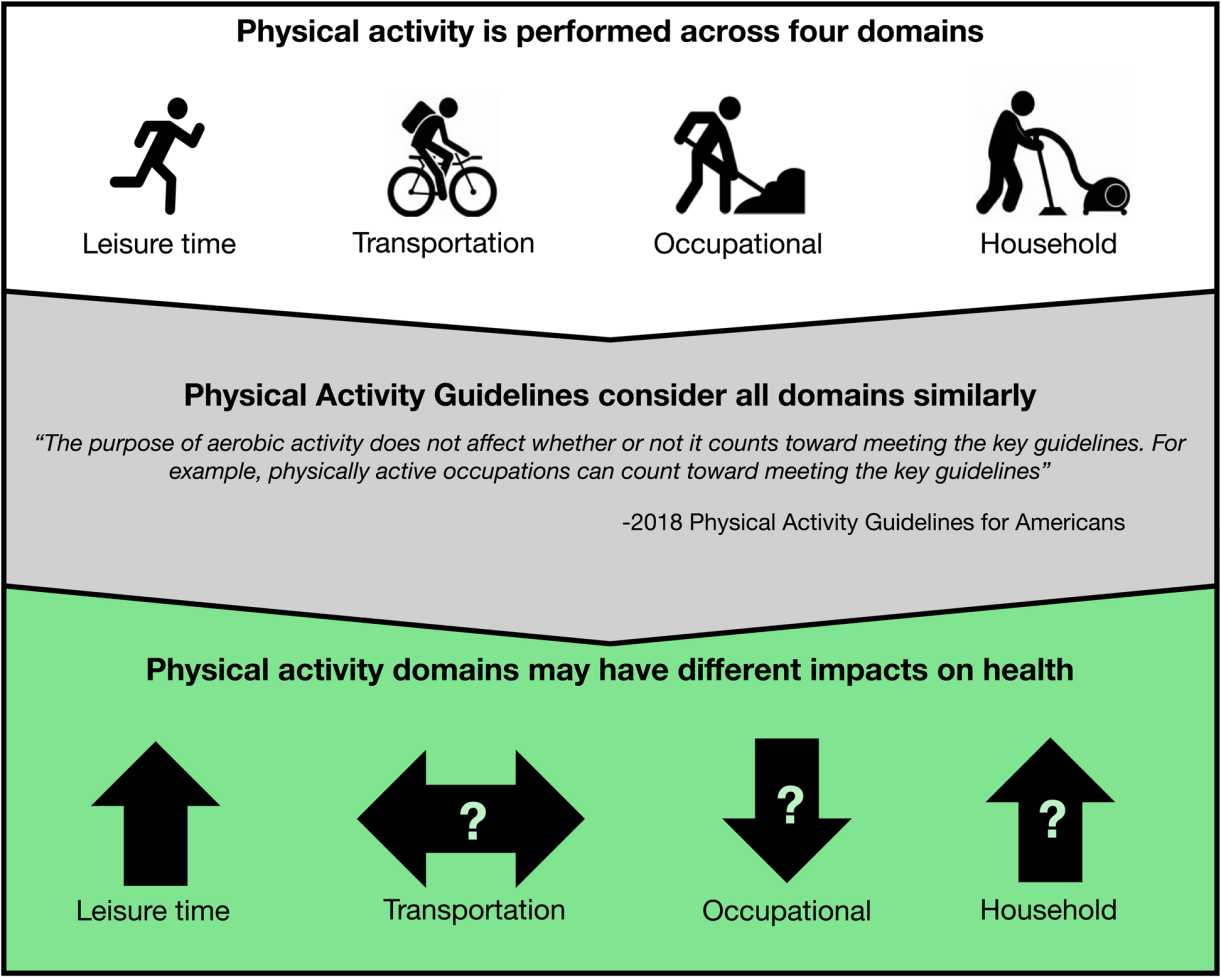
Domains of physical activity. Note: Adapted from: Quinn TD, Barone Gibbs B. J Measure Phys Behav. 2023 [3]

Physical activity is considered important for health as evidenced by existing guidelines [1, 2] and can occur across four domains: 1) leisure; 2) transportation; 3) occupational; and 4) household (Fig. 1) [3]. Recent evidence suggests that not all physical activity domains contribute equally to health outcomes. For example, it appears that workers (especially men) with high levels of occupational physical activity (OPA) may experience increased mortality as compared to those with higher levels of leisure-time physical activity (LTPA) [4, 5]. Hence, domain-specific physical activities may generate differential health outcomes, a phenomenon referred to as the “physical activity health paradox.”

In essence, the physical activity health paradox highlights the importance of considering the specific context (i.e., leisure or occupational domain) and characteristics of physical activity when evaluating its impact on health and health outcomes, including cardiovascular disorders, sickness absence, and mortality. Meanwhile, many questions remain including the need for a clear definition of the phenomenon, a rationale that supports domain-specific guidelines for physical activity, practice guidance for employers that support health-enhancing OPA, and recommendations for research and practice that address observed evidence gaps [6]. This narrative, non-systematic review, based on a symposium at the 2025 American College of Sports Medicine (ACSM) Annual Meeting, [7] aims to inform these questions by synthesizing the current epidemiological and mechanistic literature on the physical activity health paradox. We drew on prior reviews, key studies known to the authors, and targeted PubMed and Google Scholar searches, and qualitatively evaluated the strength and consistency of the evidence to highlight implications and opportunities for future work.

### Epidemiological evidence

#### Earlier evidence on OPA and health

Landmark physical activity studies such as Morris’ London bus driver and postal worker studies provided the first evidence that physical activity (in these cases OPA) was beneficial for health [8]. Evidence from these early studies suggested that workers with more physically demanding jobs (e.g., conductors on double-decker buses, postal workers delivering mail) had significantly less heart disease than their less active peers (e.g., bus drivers, desk workers [“telephonists”]). These foundational studies of OPA established the direct association between physical activity and cardiovascular health that has since been extensively confirmed. However, subsequent research on physical activity and health has more often studied LTPA rather than OPA as the exposure.

Earlier systematic review evidence suggested OPA and LTPA conferred similar health benefits. For example, Samitz et al., in their 2011 systematic review examining associations between domains of self-reported physical activity and all-cause mortality risk, found that higher volumes of both OPA (HR = 0.83, 95% CI: 0.71–0.97) and LTPA (HR = 0.74, 95% CI: 0.70–0.77) reduced the risk for all-cause mortality [9]. Sex-stratified analyses, however, found that greater OPA was only associated with reduced mortality among women, but not men. Similarly, in 2012, Li and Siegrist found that the risk for cardiovascular disease was also comparable between LTPA (RR = 0.73–0.76) and OPA (RR = 0.84–0.91), however, these researchers did not observe notable sex differences [10]. This early systematic review evidence was based on a disproportionately higher volume of studies that examined associations between LTPA and health risks, with fewer studies of OPA. For example, the meta-analysis by Samitz et al. included 41 studies (*N* = 544,056) that assessed LTPA with only six (*N* = 82,412) assessing OPA [9].

#### Contemporary evidence suggesting the physical activity health paradox

More recent systematic review evidence, based on a greater number of studies, has suggested that OPA may not confer the same health benefits as LTPA. A large systematic review and meta-analysis by Coenen et al. in 2018 (*N* = 193,696, 17 studies), observed that high vs low levels of OPA were associated with an 18% increased risk of all-cause mortality among men (HR = 1.18, 95% CI: 1.05–1.34), but with a non-statistically significant 10% reduction among women (HR = 0.90, 95% CI: 0.80–1.01) [5]. Notably, associations tended to be stronger after adjustment for LTPA, especially among men (HR = 1.25, 95% CI: 1.07–1.47).

Following this, the World Health Organization (WHO) commissioned an umbrella review to understand the health impacts of OPA and inform the development of their 2020 Physical Activity Guidelines. Cillekens et al. summarized evidence from 17 systematic reviews and found that high vs low OPA was beneficial for reducing multiple cancer outcomes, ischemic stroke, coronary heart disease, and type 2 diabetes [11]. However, high vs low OPA was also associated with an increased risk for all-cause mortality in men (reflective of Coenen et al.’s 2018 review), osteoarthritis in both sexes, and poorer sleep quality in both sexes. The risk reductions associated with OPA were similar to those for LTPA for most outcomes, with the exception of heart disease, distal colon cancer, and type 2 diabetes, where LTPA had a greater risk reduction. Yet, conclusions were limited as the certainty in the evidence from this review was mostly low-to-very low. Following this review, the WHO guideline development group concluded that there was insufficient evidence to determine whether health benefits differed by domains or types of physical activity. As a result, the current WHO guidelines suggest that all physical activity, regardless of domain, counts toward the recommended amount [2].

More recently in 2024, Kazemi et al. conducted a systematic review of 103 studies (N ~ 765 K-2.8 M [LTPA] and N ~ 53 K-730 K [OPA]) on the association between OPA, LTPA, and cardiovascular disease incidence [12]. They found that LTPA was protective for cardiovascular disease, coronary heart disease, stroke, and atrial fibrillation incidence. In contrast, they found that OPA had no significant association with these outcomes which, though not adverse, still differs from the benefits observed with LTPA. Also, the certainty of evidence was higher for LTPA than OPA.

Using data from the Active Worker Consortium, Coenen et al. conducted an individual participant data meta-analysis (*N* = 590,497), and found that, consistent with their previous review, higher levels of OPA were associated with a higher risk of all-cause mortality among men but not women [4]. They also found that LTPA was reduced the risk of all-cause mortality in both men and women.

#### Standing jobs and health risk

While research has largely examined high OPA or those who engage in physically demanding jobs, many jobs are also characterized by long periods of uninterrupted standing with little movement or sitting. This group of workers has been less studied. A study by Smith et al. is one of the most widely cited exploring the association between occupational standing and heart disease in a cohort of workers from Ontario, Canada (*N* = 7,320, 12-y follow-up) [13]. They found that, compared to occupations primarily characterized by sitting, those that primarily involved standing were associated with a significantly increased risk of heart disease (HR = 2.06, 95% CI: 1.00–4.24). This and a small number of other studies [14, 15] highlight the potential detrimental effects of prolonged occupational standing and the need for further research to study this unique OPA exposure.

#### Health risk in referent sitters

Most studies use low OPA or those that mostly engage in sitting on the job as the referent group. Importantly, occupational sitting has also been associated, though not always consistently, with higher risk of poorer health outcomes including cardiometabolic disease and premature mortality [16, 17]. A recent cohort study of workers in Taiwan (*N* = 481,688, 12.85-y follow-up) found that, compared to those who mostly did not sit, those who mostly sat at work had a higher risk for all-cause (HR = 1.16, 95% CI: 1.11–1.20) and cardiovascular disease mortality (HR = 1.34, 95% CI: 1.22–1.46) [18]. Workers who alternated sitting and non-sitting had similar (lower) risk of all-cause mortality to workers classified as mostly non-sitters. They also found that, across all workers, engaging in LTPA reduced the risk for all-cause and cardiovascular disease mortality but with a greater benefit amongst mostly sitters vs those who are more active on the job. Thus, since jobs involving a lot of sitting may carry unique health risks, it is worth noting that the health risks associated with high OPA may be underestimated when compared to a mostly sitting referent group.

#### Device-measured OPA and health

Evidence from systematic reviews has generally relied on self-reported measures of OPA. Given the improved measurement properties of device-measured physical activity (e.g., eliminates recall and response bias, ability to capture activity continuously), researchers have started to clarify associations between OPA and health using OPA measured with devices. In the ABC Study in Sweden (*N* = 807, 15-y follow-up) [19], self-reported OPA and LTPA were not associated with cardiovascular disease events: LTPA (HR = 0.74, 95% CI: 0.41–1.32); OPA (HR = 1.65, 95% CI: 0.73–3.74). Using device-measured exposures in the same cohort, LTPA was associated with a similar non-significant risk reduction (HR = 0.80, 95% CI: 0.38–1.67), yet device-measured OPA was associated with a significantly reduced risk for cardiovascular disease events (HR = 0.43, 95% CI: 0.21–0.92). In a different study of blue-collar workers in Denmark (*N* = 929, 55% male, 4-y follow-up) using device-measured physical activity, reallocating 20 min from other behaviours (sedentary, standing, light-intensity physical activity) to moderate-to-vigorous intensity physical activity during work time was associated with a 15% increase in risk of long-term sickness absence, whereas reallocating 20 min of other behaviors to moderate-to-vigorous intensity physical activity during leisure time was associated with a 20% lower risk [20]. These findings highlight the mixed evidence regarding OPA and health that remains inconclusive when device-based measures are used.

#### The role of LTPA and fitness

Current global physical activity guidelines recommend that adults achieve at least 150 min per week of moderate-to-vigorous intensity physical activity regardless of domain [2]. As a result, workers who engage in high volumes of OPA may meet or exceed the current recommendation at work, potentially reducing the likelihood that those workers seek out further opportunities to be active in their leisure time. Indeed, workers with more physically demanding jobs are found to engage in less LTPA than those in more sedentary jobs [21, 22]. This lower LTPA may also be attributed to the need for recovery from the fatigue or musculoskeletal discomfort associated with high OPA [23–25]. Regardless of the cause(s), this pattern is important as some evidence suggests that LTPA may mitigate the risks associated with high OPA by promoting cardiorespiratory fitness (henceforth referred to as “fitness”) and endurance, and by facilitating muscular adaptation, recovery, and stress relief. Systematic review and meta-analytic evidence suggests that LTPA is beneficial for all workers, but with the greatest benefit amongst those with sedentary or low OPA compared to high OPA jobs [4, 26]. A study of working adults (*N* = 5,866, 20-y follow-up) from Denmark found that, while strenuous OPA was associated with a potentially higher risk for type 2 diabetes, greater LTPA was associated with a lower risk regardless of OPA level [27]. Additionally, diabetes incidence was highest among those engaging in demanding or strenuous OPA who were also inactive during leisure time. This evidence suggests that LTPA may offset the harms for OPA. This association with LTPA may, in part, be due to greater fitness achieved through LTPA which would attenuate the cardiovascular strain of OPA. This interaction with fitness is described, in detail, within the proposed mechanisms section below.

#### Optimal worker movement profiles and health risk

Devices have provided the ability to characterize worker movement profiles (i.e., the distribution and pattern of movement behaviors such as sedentary time, physical activity, and sleep) across the 24-h time continuum and examine relationships with health. A study of 24-h movement profiles in Danish workers (*N* = 807) using thigh-worn accelerometry suggests that, compared to those who have a more balanced distribution of movement behaviours in work and leisure, those who are more active at work but less during leisure and those who are always sedentary (work and leisure) had lower fitness [28]. Interestingly, those who were more active at work but less during leisure had lower blood pressure.

Similarly, a study of 8,909 working adults from the Canadian Health Measures Survey used cluster analysis to identify movement profiles based on accelerometer data and assess their association with 10-year cardiovascular disease risk [29]. Workers who accumulated physical activity throughout the day, including both work and leisure hours, were found to have more optimal cardiovascular risk profiles. In contrast, those who accumulated physical activity only during work hours did not have a reduced risk. These findings suggest that workers who engage in a more balanced day, including OPA and LTPA, have better health outcomes.

#### Summary of epidemiological evidence and future opportunities

**Fig. 2:**
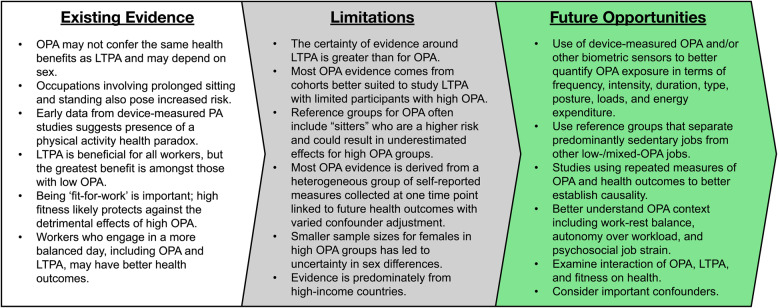
Summary of epidemiological evidence and future opportunities. Abbreviations: LTPA = leisure-time physical activity, OPA = occupational physical activity

A summary of the epidemiological evidence including current limitations and areas in need of future research is provided in Fig. 2. The nature and extent of the physical activity health paradox remains unclear given limitations of the available research. Most of the evidence to date is of lower certainty and quality, having relied predominantly on self-reported LTPA and OPA assessed at baseline and linked to outcomes at follow-up, with confounder adjustment including a variable combination of sociodemographics, work factors, and other health behaviours [30]. OPA measurement methodology is inconsistent, with OPA used as an umbrella term to describe a range of physical activity exposures during work including energy expenditure, postures, loads, and job types [31]. Many of these measures fail to capture differences in the intensity, frequency, and duration of activities within broad job categories. Additionally, when device-based physical activity assessments are included, the methods and samples are often optimized for quantifying LTPA rather than OPA-specific movement patterns (e.g., lifting), which could lead to error and bias in OPA exposure estimates [32–34]. This lack of standardization and precise characterization of OPA limits our ability to draw robust conclusions and may partly explain why OPA sometimes has negative or null relationships with health outcomes. Future research would benefit from a clearer distinction between these features of OPA to better understand the mechanism behind associations with health and to design effective intervention strategies. It is also possible that the healthy worker effect is present in some studies, whereby those who are employed (and continue to be employed) in physically demanding jobs tend to be healthier than the general population, and those who are ill or have chronic conditions may be less likely to be employed in such jobs.

Beyond measurement issues, many large-scale cohorts and surveillance systems utilized to study OPA have enrolled predominantly higher-educated participants with white-collar jobs, limiting variability in OPA due to few participants with very high-OPA jobs [4, 35, 36]. In such samples, the “true” dose–response between OPA and cardiovascular risk may be only partially observed. For example, if the underlying association is U-shaped as some studies suggest [34–36], with harm concentrated at very high cumulative OPA, underrepresentation of the highest OPA workers will tend to produce flatter or even protective associations. This challenge may be even greater in device-based studies, which often include a more selected subset of participants [22, 37]. Lastly, the available studies predominately examine mostly higher-income countries from select geographic regions (e.g. Scandinavia, Israel, Spain) where OPA exposure and workplace regulations may differ from the rest of the world [38].

Future research could consider: (1) incorporating device-measured OPA alongside other biometric sensors such as heart rate to be able to better quantify the intensity, frequency, and duration of OPA; (2) purposeful sampling of workers with high-OPA jobs in cohorts examining OPA and health risk; (3) dimensions of physical job demands including posture, loads, and energy expenditure; (4) using referent groups that separate predominantly sedentary jobs from other low- or mixed-OPA jobs, to limit the impact of the known risk from prolonged sitting on the effects of high OPA; (5) important contextual information including work-rest balance, autonomy or control over the workload, and psychosocial job strain that may moderate or amplify the health effects of OPA; (6) including repeated measures data on OPA, outcomes, and important confounding factors to better assess changes and effects; (7) examining the interaction between OPA and LTPA and fitness on health; and (8) including important confounders such as age, gender, socioeconomic status, work conditions (including stress), health behaviours (e.g., diet, alcohol, smoking), anthropometric data (e.g., waist circumference), medication, and competing health conditions.

### Proposed mechanisms

Several mechanisms have been proposed to explain why OPA might be uniquely harmful or render less benefit to cardiovascular and overall health as compared to LTPA, which is known to be beneficial. Unlike LTPA, OPA is typically performed at lower intensities, over prolonged durations, and with insufficient opportunities for rest and recovery [39–41]. This unique pattern of physical activity likely fails to stimulate beneficial cardiorespiratory adaptations and may, conversely, impose chronic cardiovascular strain. With repeated, long-term exposure, this strain could be detrimental to cardiovascular health.

The current physical activity health paradox hypothesis posits that the nature and pattern of OPA, characterized by repetitive, low-intensity movements sustained across the workday, elicits acute increases in cardiovascular strain (i.e., elevated heart rate and blood pressure) sustained throughout work [42]. Over time, this chronic strain may catalyze systemic inflammation and vascular damage, contributing to adverse cardiovascular remodeling and increased disease risk. Furthermore, factors such as low fitness and high work-related stress appear to amplify these physiological responses, exacerbating the health risks associated with OPA.

This section synthesizes the current mechanistic evidence examining these proposed pathways and defines evidence gaps for future exploration.

#### Cardiovascular strain

OPA often involves prolonged, low-to-moderate intensity activities performed throughout the workday, and frequently with insufficient opportunities for rest and recovery. Unlike LTPA, which is typically performed at higher intensities in shorter bouts with adequate recovery, OPA can lead to elevated resting blood pressure and sustained elevations in heart rate and blood pressure across the 24-h period.

Converse to the beneficial impacts from LTPA, OPA was not associated with reduced resting blood pressure or hypertension risk in the population-based Lifelines Cohort (*n* = 125,402; median age = 45; 40.5% male; 27.7% low education) [43]. Similarly, Ohlin et al. observed that OPA was associated with higher resting blood pressure in a cross-sectional study of 241,176 Swedish male construction workers [44]. Li et al. also observed that high levels of OPA were associated with an increased risk of developing new-onset hypertension in a large prospective cohort study of over 9,000 Chinese adults, emphasizing the potential long-term cardiovascular risks of chronic occupational strain [45].

OPA has also been associated with increased ambulatory blood pressure and heart rate responses. A study of Belgian workers engaging in high OPA exhibited significantly higher 24-h systolic blood pressure compared to their low OPA counterparts, even after accounting for leisure-time activity [46]. Similarly, Coenen et al. found that heart rate reserve was consistently higher during OPA tasks compared to LTPA of similar intensity, suggesting an elevated relative cardiovascular strain at work [47]. In our own work, we observed that workers exhibited higher 24-h heart rate and blood pressure on workdays compared to non-workdays, indicating a greater cumulative cardiovascular load associated with OPA [48]. These data support the idea that daily OPA increases the cardiovascular load on the heart, which could increase cardiovascular disease risk.

Furthermore, greater cardiovascular load induced by OPA may not be limited to work hours but could extend into nighttime recovery periods. Hallman reported that higher OPA was associated with elevated heart rate and reduced heart rate variability (HRV) during sleep, reflecting an impaired autonomic recovery [49]. Our within-subjects study of 19 workers observed similar patterns where nocturnal HRV was reduced on workdays with high OPA compared to non-workdays [48]. Such nocturnal autonomic dysfunction may be a critical pathway linking OPA to long-term cardiovascular risk [50]. Experimental evidence further supports these findings. In a randomized controlled trial, Korshøj et al. (2017) implemented a workplace aerobic exercise intervention among cleaners, demonstrating that moderate-intensity aerobic exercise lowered 24-h ambulatory blood pressure. These data suggest that improving fitness may mitigate OPA-induced blood pressure elevations [51].

#### Vascular impacts

In addition to cardiovascular strain, chronic OPA has been associated with adverse vascular adaptations that may underlie its association with cardiovascular disease. Arnold et al. (2021) reported that high OPA was linked to increased arterial stiffness, an established subclinical precursor of cardiovascular disease [52]. Longitudinal data from the CARDIA study revealed that sustained high OPA exposure over 25 years was associated with adverse changes in left ventricular structure and function, providing evidence for maladaptive cardiac remodeling as a potential pathway linking OPA to cardiovascular disease [53]. Supporting these vascular mechanisms, Climie et al. found that higher OPA was associated with impaired neural baroreflex sensitivity, a critical marker of autonomic cardiovascular dysregulation, suggesting that prolonged OPA strain may blunt the body’s ability to maintain stable blood pressure control and vascular homeostasis [54].

#### Inflammation

OPA has also been associated with biomarkers indicative of systemic inflammation. A recent narrative review by Jordakieva et al., observed that both sedentary and high-intensity OPA, particularly involving heavy lifting, were associated with elevated systemic inflammation and increased cardiovascular and cancer mortality risk [55]. Supportive of these conclusions, Feinberg et al. observed elevated inflammatory markers among workers with high OPA [56]. Similarly, OPA, but not LTPA, was associated with elevated high-sensitivity C-reactive protein in a large Korean cohort, suggesting a pro-inflammatory effect of OPA that may contribute to adverse cardiovascular outcomes. Together, these findings suggest that OPA may disrupt the body’s ability to control inflammation, leading to a persistent, elevated inflammation that increases long-term risk for heart disease and other chronic conditions.

#### Worker fitness

Fitness is one of the strongest predictors of future health and longevity [57]. High volume cardiovascular strain may be an underlying driver of the physical activity health paradox and greater fitness achieved through LTPA may attenuate this strain and the related risks of high OPA [58]. The ‘fit-for-work’ principle emphasizes the importance of having sufficient fitness to meet the physical demands of a job. Unfortunately, worker fitness appears to be declining with the greatest reductions among blue-collar workers [59, 60]. Paradoxically, high OPA levels appear to be associated with lower fitness in cross-sectional and longitudinal analyses [20, 61]. A study of 50,000 Swedish workers found that most high OPA workers had insufficient fitness to meet the physical demands of the job [62].

Ketels et al. combined data from five European cohorts (*N* = 9,922 men, 26-y follow-up) to examine the interaction between self-reported OPA and objectively measured fitness [63]. They found that greater fitness protected against the detrimental associations between higher OPA and cardiovascular mortality, but not all-cause mortality. Mechanistic studies on the topic suggest an explanation where workers with low fitness may have exaggerated cardiovascular response to OPA due to the high cardiovascular demand of the work tasks relative to their cardiorespiratory capacity [47, 48, 64]. However, somewhat contrary to this explanation, a randomized controlled trial found that improving fitness of cleaners with high levels of OPA resulted in adverse impacts to heart rate and blood pressure [65]. These studies suggest that worker fitness may play a role in understanding the impact of OPA on health and warrants further research. As the ‘fit-for-work’ principle suggests, it may be that high fitness protects workers with physically demanding jobs from adverse cardiovascular outcomes. However, the underlying mechanistic work is very limited and must be expanded upon moving forward.

#### Psychosocial stress

Beyond the physical demands of OPA, the workplace cultural context, such as norms, expectations, and limited worker autonomy, may introduce additional psychosocial stressors that further exacerbate cardiovascular strain, vascular effects, and inflammation, compounding the effects of OPA to further elevate disease risk through both physiological and behavioral pathways. Previous work has demonstrated that psychological stress raises ambulatory blood pressure independently [66]. When examined as a modifier to the associations between cardiovascular strain and OPA, our recent study found that the combination of high OPA and elevated work stress significantly exaggerated 24-h ambulatory heart rate and blood pressure responses, exacerbating the cardiovascular load [64]. Furthermore, some work suggests that high work stress may increase risk for coronary heart disease, underscoring the need to consider psychosocial factors alongside physical work demands which could interact to further increase risks [67]. It should lastly be acknowledged that, unlike LTPA, OPA is often completed without autonomy over the activities performed and without an explicit intention to positively impact health [68]. Salvo et al. describe this pattern as “necessity-based” physical activity, in contrast to discretionary, “choice-based” LTPA, and argue that necessity-driven movement is more likely to be experienced as compulsory and constrained by broader social and economic conditions [53]. While research is limited on the topic, the lack of autonomy and/or intention may contribute to additional psychological or physiological stress adverse to health.

#### Summary of mechanistic research and future opportunities

**Fig. 3:**
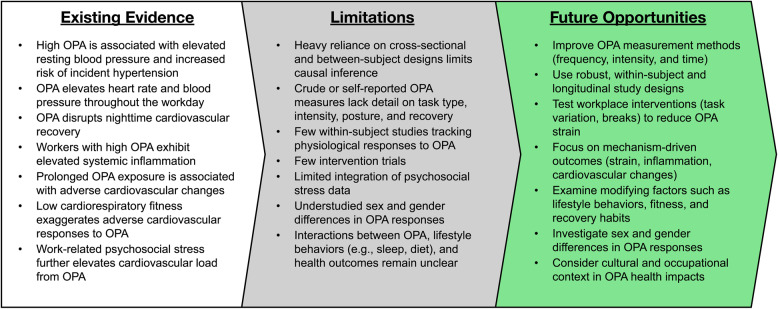
Summary of mechanistic evidence and future opportunities. Abbreviations: LTPA = leisure-time physical activity, OPA = occupational physical activity

A summary of the current mechanistic evidence, associated limitations, and future opportunities is presented in Fig. 3. While more evidence is needed to fully understand the proposed explanatory mechanisms, the available data provides a plausible biological basis for the physical activity health paradox.

However, several limitations in the current mechanistic literature must be acknowledged. Much of the existing evidence is derived from observational cross-sectional and (fewer) longitudinal cohort study designs which are vulnerable to residual confounding and limit causal inference. Few studies have rigorously examined within-subject changes or leveraged mechanistic intervention designs. Furthermore, assessments of OPA are often crude, lacking detailed characterization of activity type, posture, intensity, and recovery opportunities. Future research should prioritize multidimensional OPA measurement approaches, more rigorous study designs to examine mechanisms, and focus on mechanism-informed outcomes wherever possible.

### Challenges and opportunities for research and practice

Unlike the established health benefits from LTPA, the observed effects of OPA on health remain mixed. Though some, and mostly older, studies have found that OPA is associated with the expected health benefits of physical activity, more recent research suggests that OPA either has no impact on health or may even be harmful. Emerging studies identify cardiovascular and other mechanisms that could reasonably explain why OPA may not be as beneficial as LTPA, strengthening the case for plausibility and causal inference. Yet, limitations such as poor characterization and measurement of OPA and minimal experimental data reduce the strength of the evidence supporting the physical activity health paradox. Addressing this uncertainty is important for public health as the current, domain-agnostic guidelines allow for weekly physical activity recommendations to be accumulated in any domain (including OPA). This may be under-serving individuals who accumulate high levels of OPA, because such individuals may be “meeting guidelines” through OPA but getting fewer benefits than if they were accumulating the same volume of activity as LTPA. Importantly, these individuals are overrepresented in low income and racial or ethnic minority populations who are already at increased cardiovascular disease risk [69]. Improved understanding and awareness of the physical activity health paradox may help to reduce existing health inequities, as well as provide impactful and individualized pathways towards health for people from all socioeconomic groups, including those with jobs requiring high-OPA exposure. Given the possibility and importance of the paradox coupled with the remaining scientific uncertainty, where do we go from here?

#### Implications for public health guidelines

First, public health practitioners must continue to evaluate the available evidence on OPA and health for public health promotion guidance and programming. Since the phrase “health paradox” was coined in 2012, research seeking to disentangle this complicated research question has accelerated [70]. Continued evaluation of the paradox as research becomes available is critical. Similar to the WHO’s process prior to releasing the 2020 Guidelines, the United States Department of Health and Human Services included OPA as a key topic in their call-to-action in preparation for the anticipated 2028 update of the Physical Activity Guidelines for Americans [71]. Such large-scale, expert evaluation of the strength of evidence supporting the physical activity health paradox will expose the most important research gaps, direct researchers to fill those gaps, and facilitate translating research into practice. It is possible that further expert review may remain inconclusive regarding whether OPA is harmful, has no effect on health, or is beneficial. Yet, based on the current evidence and recognizing its lower certainty, it seems likely that OPA is less beneficial than LTPA. This would support guidelines to consider general domain-specific statements like, ‘*Engaging in leisure physical activities such as walking, sports, swimming, or gardening may provide the most health benefits’ or ‘A balanced day that includes leisure-time physical activities, is important for achieving good health’.* At the least, it is our responsibility to clearly communicate up-to-date understanding of the evidence to the public while researchers continue the pursuit to clarify the existence and extent of the physical activity health paradox.

#### Implications for employers and workers

Some pragmatic considerations for employers and workers are also reasonable given the available evidence. This approach is further justified since the workplace is a useful and important setting for health promotion, public health, and the mutual employee-employer benefits gleaned from promoting worker health (for more, see Pronk’s elegant discussion from 2024 in the *British Journal of Sports Medicine*) [6]. For example, most available investigations of the physical activity health paradox find that the negative effects of OPA on health are attenuated or abolished with higher fitness. Therefore, workplace health promotion programming such as exercise training during work or leisure time to enhance fitness in workers with lower fitness levels and high OPA demands is likely appropriate. To do this, engaging stakeholders including workers, health plans, and employers is a best practice approach to optimize feasible, acceptable, and sustainable workplace health promotion programs in the context of jobs with high OPA.

#### Implications for researchers

The uncertainty regarding the physical activity health paradox is a testament to the unique methodological challenges for studying OPA. A primary challenge to studying and synthesizing the current literature on this topic is that the definition and, relatedly, best practice measurement approaches have not been established. For the sake of comparison, LTPA has been extensively characterized and studied across aspects of the FITT (frequency, intensity, time, type) principles. Research across aspects of FITT for LTPA has yielded many important conclusions that inform public health guidelines. For example, vigorous-intensity aerobic activity provides health benefits at shorter durations than moderate-intensity aerobic activity and that aerobic and muscle-strengthening activity yield complimentary health benefits such that guidelines promote engaging in both for optimal health [1, 2]. In contrast, OPA has to date been largely classified as a single exposure [11, 72]. These complexities make defining and identifying the potentially harmful element(s) of OPA more challenging. Still, Lord Kelvin’s famous quote, “*if you cannot measure it, you cannot improve it,*” certainly applies. Accurate and comprehensive measurement of the multiple dimensions of OPA is needed to advance scientific inquiry, including but not limited to: (1) sensors to measure the frequency, intensity, and time dimensions (FIT) of upper and lower body movement during OPA (e.g., accelerometers, heart rate monitors, multi-sensors); (2) time-use diaries or ecological momentary assessment to measure OPA type as well as psychological aspects of OPA including autonomy and perceived stress; (3) physiological responses to OPA (e.g., stress by cortisol, continuous glucose monitoring, and continuous heart rate and blood pressure); and (4) temporal patterning and compounding effects of OPA, LTPA, and rest across days, weeks, or longer timeframes. Given the complexity of OPA exposure, future research will need to leverage advanced data analytics and machine learning methods to identify nuanced OPA exposure profiles and relate them to cardiovascular and metabolic outcomes to better understand health impacts.

Designing rigorous research clarifying the health impacts of OPA has challenges beyond measurement. Observational studies are vulnerable to the potential for residual confounding, which may overestimate the harms of OPA due to co-occurrence of socioeconomic factors or health behaviors linked to poorer health outcomes. At the same time, reverse causation or confounding by indication may underestimate effects as sicker workers change to lower OPA jobs or leave the workforce. Classic analytic strategies such as restriction or stratification on key characteristics (e.g., by smoking), longitudinal designs, and careful covariate adjustment may in part address these concerns for bias. Though randomized controlled trials would be most rigorous, the ethical and logistical barriers to conducting experimental research of OPA exposures make such gold standard approaches more difficult. Again, using the example of LTPA, exercise training is generally accepted as health enhancing and encompasses a small portion of the day, facilitating experimental manipulation in randomized trials. On the other hand, while trials have examined promoting LTPA in workers with high OPA [51, 73] and intervening on OPA intensity [74], attempts to experimentally manipulate OPA are ethically ambiguous given the potential harm of OPA. OPA trials are also less feasible since OPA exposure is more complex, takes up most of the day, and would likely require workers to alter their assigned work tasks and/or schedule. Employers may see such research as a distraction and are often risk averse to exposing workers to potential harms of OPA performed under their purview, regardless of the trial’s objectives. The unique challenges to conducting rigorous research on OPA and health will necessitate ingenuity by the scientific community to get to the bottom of the physical activity health paradox. Some ideas that could move science forward include: (1) natural experiments where OPA exposure is changed and historical or unchanged group comparisons are available; (2) within-subject designs that leverage variability in OPA exposure; or (3) mitigation trials where aspects of OPA can be manipulated (e.g., add standardized rest breaks or increase task variability). Concurrently, more research on the proposed mechanisms of the physical activity health paradox will be synergistic to focus research efforts on aspects of OPA exposure that are most plausibly leading to poor health outcomes.

## Conclusion

Whether we are active at work or during leisure may matter when it comes to the health benefits that we can expect to earn. The physical activity health paradox evidence suggests that domain-specific physical activity guidance to support public health should be considered. Guidelines for healthy physical activity during and away from work also need to be viewed through a health equity lens as high and sustained intensity OPA is more common in specific subpopulations, such as low income and racial or ethnic minority populations, who are already often at higher health risk. Recommendations for research and practice also need to recognize domain-specific impacts, measurement challenges, and the inherent complexity of the observed paradox.

## Data Availability

No datasets were generated or analysed during the current study.

## Abbreviations

LTPA: Leisure-time physical activity
OPA: Occupational physical activity
